# Impact of exudative diathesis induced by selenium deficiency on LncRNAs and their roles in the oxidative reduction process in broiler chick veins

**DOI:** 10.18632/oncotarget.14971

**Published:** 2017-02-01

**Authors:** Changyu Cao, Ruifeng Fan, Jinxin Zhao, Xia Zhao, Jie Yang, Ziwei Zhang, Shiwen Xu

**Affiliations:** ^1^ Department of Veterinary Medicine, Northeast Agricultural University, Harbin 150030, P. R. China; ^2^ Key Laboratory of Animal Cellular and Genetic Engineering of Heilongjiang Province, Northeast Agricultural University, Harbin 150030, P. R. China

**Keywords:** exudative diathesis, selenium deficiency, long noncoding RNA-seq, oxidative reduction process, broiler chick vein

## Abstract

Selenium deficiency may induce exudative diathesis (ED) in broiler chick, and this damage is closely related to oxidative damage. Long noncoding RNA (LncRNA) can regulate the redox state *in vivo*. The aim of the present study was to clarify the LncRNA expression profile in broiler veins and filter and verify the LncRNAs related to oxidative damage of ED. This study established an ED model induced by selenium deficiency and presented the expression and characterization of LncRNAs in normal and ED samples. A total of 15412 LncRNAs (including 8052 novel LncRNAs) were generated in six cDNA libraries using the Illumina Hi-Seq 4000 platform. 635 distinct changes in LncRNAs (up-regulated fold change > 1.5, down-regulated fold change < 0.67 and differentially expressed LncRNAs) were filtered. Gene ontology enrichment on LncRNAs target genes showed that the oxidative reduction process was important. This study also defined and verified 19 target mRNAs of 23 LncRNAs related to the oxidative reduction process. The *in vivo* and vitro experiments also demonstrated these 23 LncRNAs can participate in the oxidative reduction process. This study presents LncRNAs expression profile in broiler chick veins for the first time and confirmed 23 LncRNAs involving in the vein oxidative damage in ED.

## INTRODUCTION

Long noncoding RNAs (LncRNAs) are a course of transcripts longer than 200 nucleotides that do not encode proteins. Increasing evidence demonstrates the important roles of LncRNAs in regulating many biological processes including transcription regulation [1], post-transcriptional processing [2], and subcellular trafficking [3] by mechanisms that are not yet fully understood. Studies have found the transcripts of LncRNAs in humans, pigs and mice [4–6]. LncRNAs are emerging as regulators of vascular function in health and disease. It is increasingly important to rapidly identify LncRNAs that are implicated in vascular disease [7]. LncRNAs can also regulate redox state *in vivo*. Sun reported SCAL1 is an LncRNA that shows increased levels as a part of the oxidative stress response in lung carcinogenesis [8], Puthanveetil reported MALAT1 is an initiator of oxidative stress [9].

Increased susceptibility to oxidative stress and the resulting injury are thought to participate in the onset and progression of many pathophysiological processes such as endothelial dysfunction, hypertension, diabetes, inflammatory, and metabolic diseases [10–14]. Many studies have demonstrated the roles of Se in regulating the oxidative status [15, 16]. Se deficiency may induce oxidative stress in many tissues including brain, intestine and muscles [17–19]. Experimental evidence indicates that increased oxidative stress and relative oxidative damage are mediators of vascular pathologies [20, 21]. We previously showed that Se deficiency may influence the oxidative state in both arteries and veins, with veins being more sensitive than arteries [22, 23]. Broiler chicks are susceptible to Se deficiency diseases, especially exudative diathesis (ED) [24, 25]; it is closely related to vascular oxidative damage [23]. To understand the transcript LncRNAs in normal broiler chick vein and how LncRNA changes regulate the oxidative reduction process in ED, this study established the ED model, detected the LncRNA expression profile in broiler chick vein, and applied bioinformatics methods to distinguish between oxidative reduction-related LncRNA and the target mRNA. We also proofed the sequencing results in vein tissue and vein endothelial cell (VEC), detected the LncRNA expression profile in broiler vein, selected LncRNA related with oxidative reduction process, and provided references for a thorough study of the vein damage induced by Se deficiency.

## RESULTS

### Observation of animals

**Figure 1:**
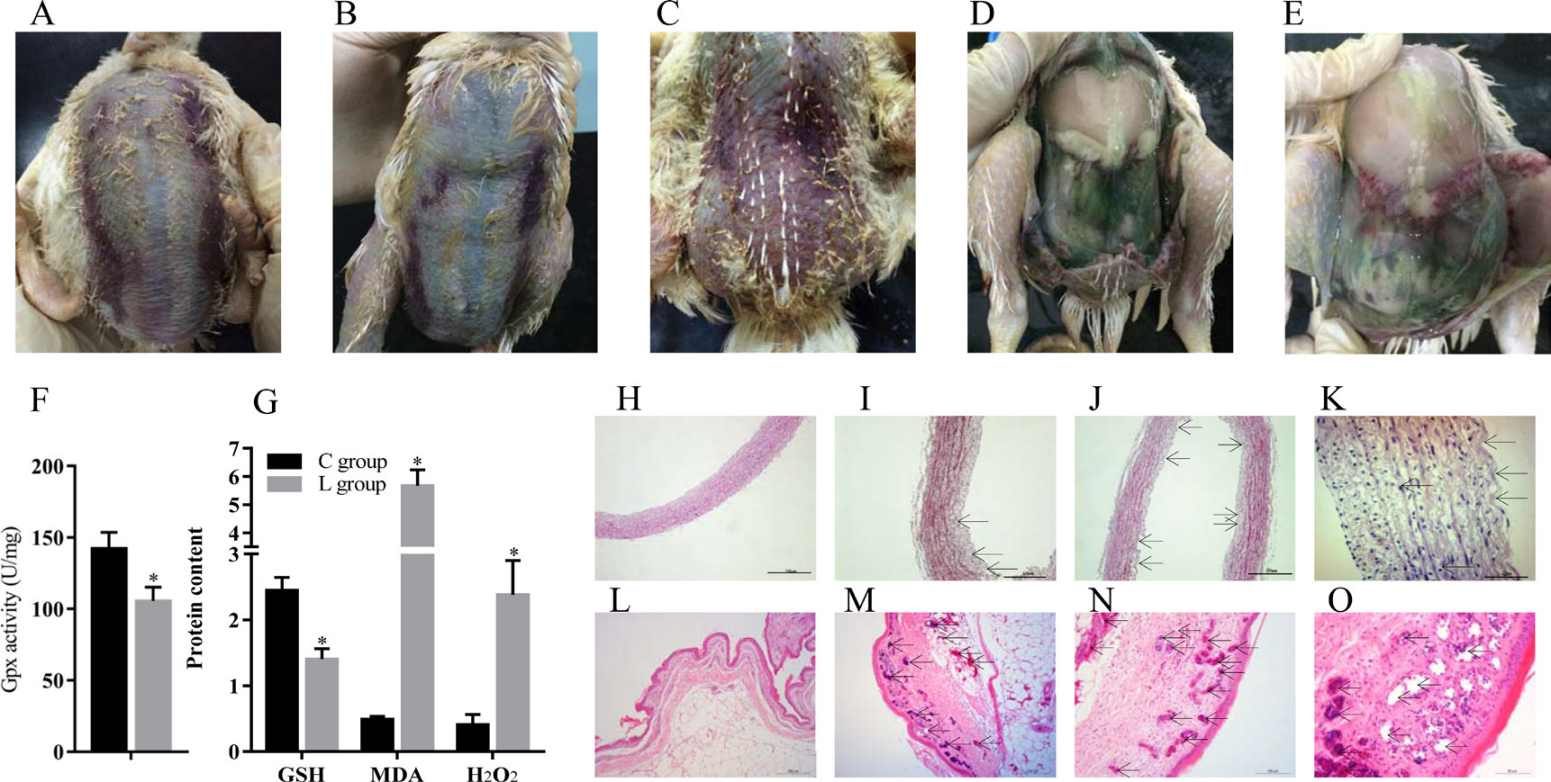
Se deficiency induce exudative diathesis and HE staining for broiler chick veins and skins (**A**–**C**) The typical exudative diathesis; (**D**, **E**) Necropsy photograph of Se deficient broilers; (**F**) Effect of Se deficiency on GPx; (**G**) Effect of Se deficiency on GSH, MDA and H_2_O_2_. The unit of GPx, GSH, MDA and H_2_O_2_ were U mg^−1^, mg prot^−1^, nmol·mgprot^−1^ and mmol·gprot^−1^; (**H**) The histopathological analysis in vein of control group; (**I**–**K**). The histopathological analysis in vein of Se deficiency group, the arrows in black point to the location of the lesion; (**L**) The histopathological analysis in skin of control group; (**M**–**O**) The histopathological analysis in skin of Se deficiency group, the arrows in black point to the location of the lesion. Each value represented the mean ± S.D. of three individuals. *P < 0.05 versus control group.

We observed broiler chicks in the Se-deficient group showed typical Se deficiency symptoms, including ED in parts of loose skin and muscular hemorrhage (Figure 1A, 1B, 1C). Significant exudation and varying degrees of bleeding were also visible at necropsy (Figure 1D, 1E).

### The effects of dietary Se on the antioxidant related factors in broiler vein

4 antioxidant related factors, including Gpx, GSH, MDA and H_2_O_2_ were affected by dietary Se (Figure 1F and 1G). Compared with the broiler veins in the C group, the vein Gpx activity as well as the content of GSH was decreased (*P* < 0.05) by dietary Se deficiency; however, the contents of MDA and H_2_O_2_ were increased (*P* < 0.05).

### Pathological and histopathological changes in broiler vein and skin

The change of skin and vein in the Se deficiency group is shown in Figure 1H–1O. Histopathology changes showed blood vessel wall thickening, fibrinoid degeneration, endothelial cells missing from the intimal layer, disorder in the myofibrillae, and inflammatory cell infiltration in the broiler veins of the Se deficiency group (Figure 1I, 1J, 1K). Histopathology changes also included vascular proliferation, expansion and congestion in dermal tissue. A large number of inflammatory cell infiltrations were visible in the dermis near the epidermis. Small blood vessel congestion was also found in skin (Figure 1M, 1N, 1O).

### Overview of RNA sequencing

To identify LncRNAs expression in Se deficiency and BD treated broiler vein, six cDNA libraries were constructed and sequenced using the Illumina Hi-Seq 4000 platform. A total of 52298811, 49471658, 47484196, 46510957, 45206148 and 45343151 raw reads were generated, respectively. The GC content of each library was 48.02%, 47.49%, 46.69%, 46.13%, 47.25% and 45.72%, respectively. In addition, 50013920 (95.63%), 47448060 (95.91%), 46291224 (97.49%), 44859949 (96.45%), 43555507 (96.35%) and 43891272 (96.8%) clean reads remained and were used in the following analysis after discarding those reads with adapters, poly-N > 10% and any other possible contaminants (Table 1). Subsequent analysis was based on only the clean reads.

**Table 1:** Data output quality conditions

Sample name	Raw reads	GC content(%)	Clean reads	Clean reads/Raw reads(%)
C-1	52298811	48.02	50013920	95.63%
C-2	49471658	47.49	47448060	95.91%
C-3	47484196	46.69	46291224	97.49%
L-1	46510957	46.13	44859949	96.45%
L-2	45206148	47.25	43555507	96.35%
L-3	45343151	45.72	43891272	96.80%

### Identification and profiling of LncRNAs in broiler vein

**Figure 2:**
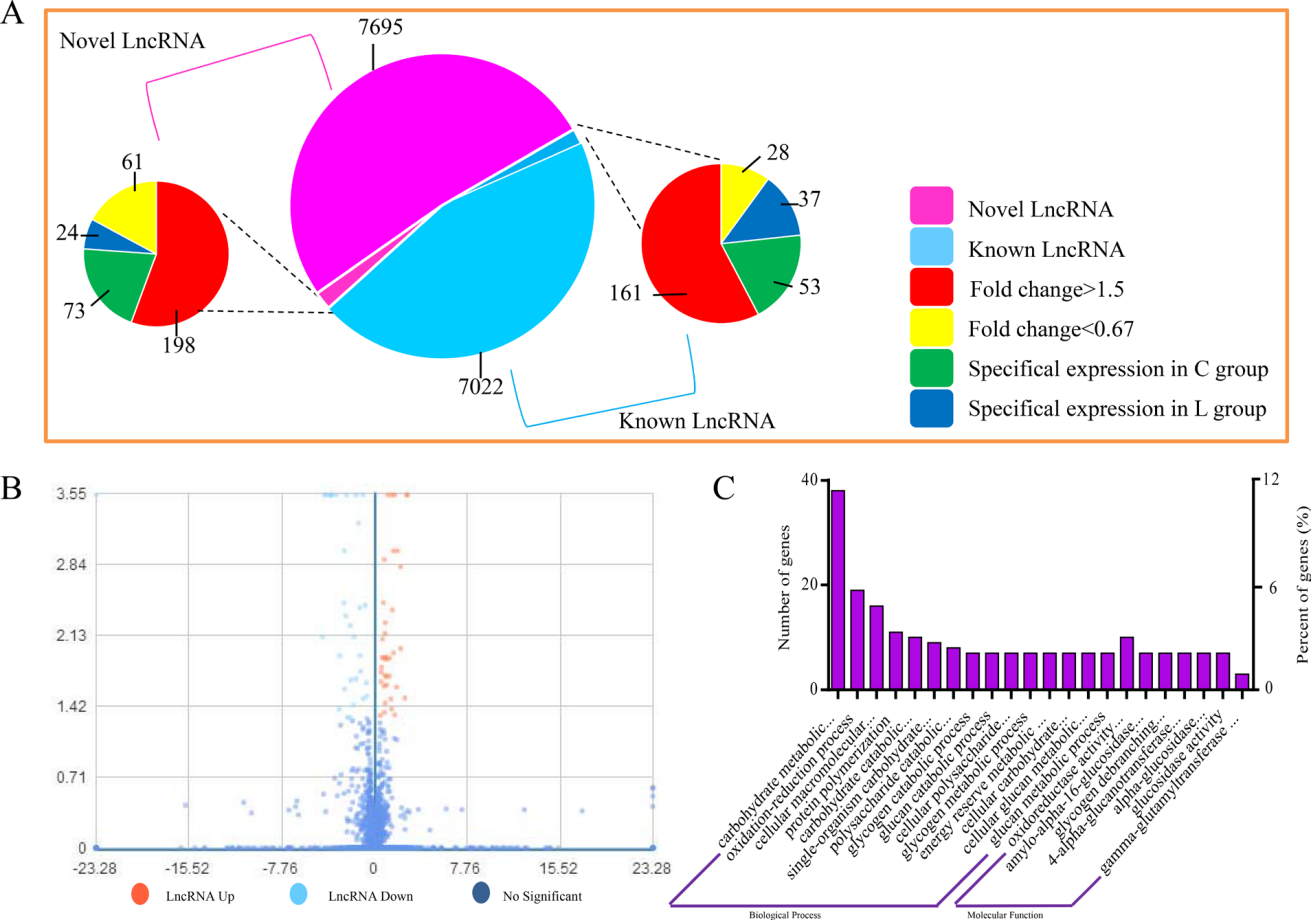
Expression of LncRNAs during Se deficiency process in broiler chick vein (**A**) Volcano map for different LncRNA. X coordinate indicated fold change, Y coo rdinate indicated significance, red dot were up regulated LncRNA, light blue dot were down regulated LncRNA, blue dot were not different LncRNA. (**B**) Expression patterns. (**C**) GO enrichment for LncRNA target genes. X coordinate was the GO term of GO enrichment. The Y coordinate was the number of candidate genes from each term. Three types of GO enrichments including biological process and molecular function.

Whether coding potential is present or not is the key condition to judge whether the transcript is an LncRNA. We combined the mainstream coding potential analysis methods to filter the transcripts. A total of 15412 LncRNAs were identified, and from an intersection of the analysis results of CPC, CNCI, pfam and phyloCSF, 8052 LncRNAs were discovered for the first time. The expression level of LncRNA transcripts in broiler vein was estimated by FPKM. A volcano map for different transcript LncRNA is shown in Figure 2A. A total of 359 up-regulated (Fold change > 1.5) and 86 down-regulated (Fold change < 0.67) LncRNAs were detected in broiler vein (*P* < 0.05). 126 LncRNAs were only detected in the L group, while 61 LncRNAs were only detected in the C group (Figure 2B).

### Prediction of the function of LncRNAs

Most of the LncRNAs in current databases have not yet been functionally annotated. Thus, the prediction of their functions is based on the functional annotations of their related cis target mRNAs. We defined potential cis-regulated target genes as protein-coding genes within 10 kb and 100 kb in genomic distance from the differentially expressed LncRNA.

### Enrichment analysis of nearest neighbor genes of LncRNAs

GO term analysis was executed for exploring the transcripts functions. Biological process, cellular component and molecular function in GO term reflects the distribution of transcripts directly. In this study, 22 GO terms were significantly enriched (*P* < 0.05), mainly involved in carbohydrate metabolic processes (GO: 0005975), oxidation-reduction processes (GO: 0055114), cellular macromolecular complex assembly (GO: 0034622), protein polymerization (GO: 0051258) and carbohydrate catabolic processes (GO: 0016052) (Figure 2C). GO term analysis showed that these target genes of differentially expressed LncRNAs were enriched in pathways related to ED disease such as oxidative stress.

### LncRNA levels of genes related to oxidative stress in broiler vein by RNA-seq

**Figure 3:**
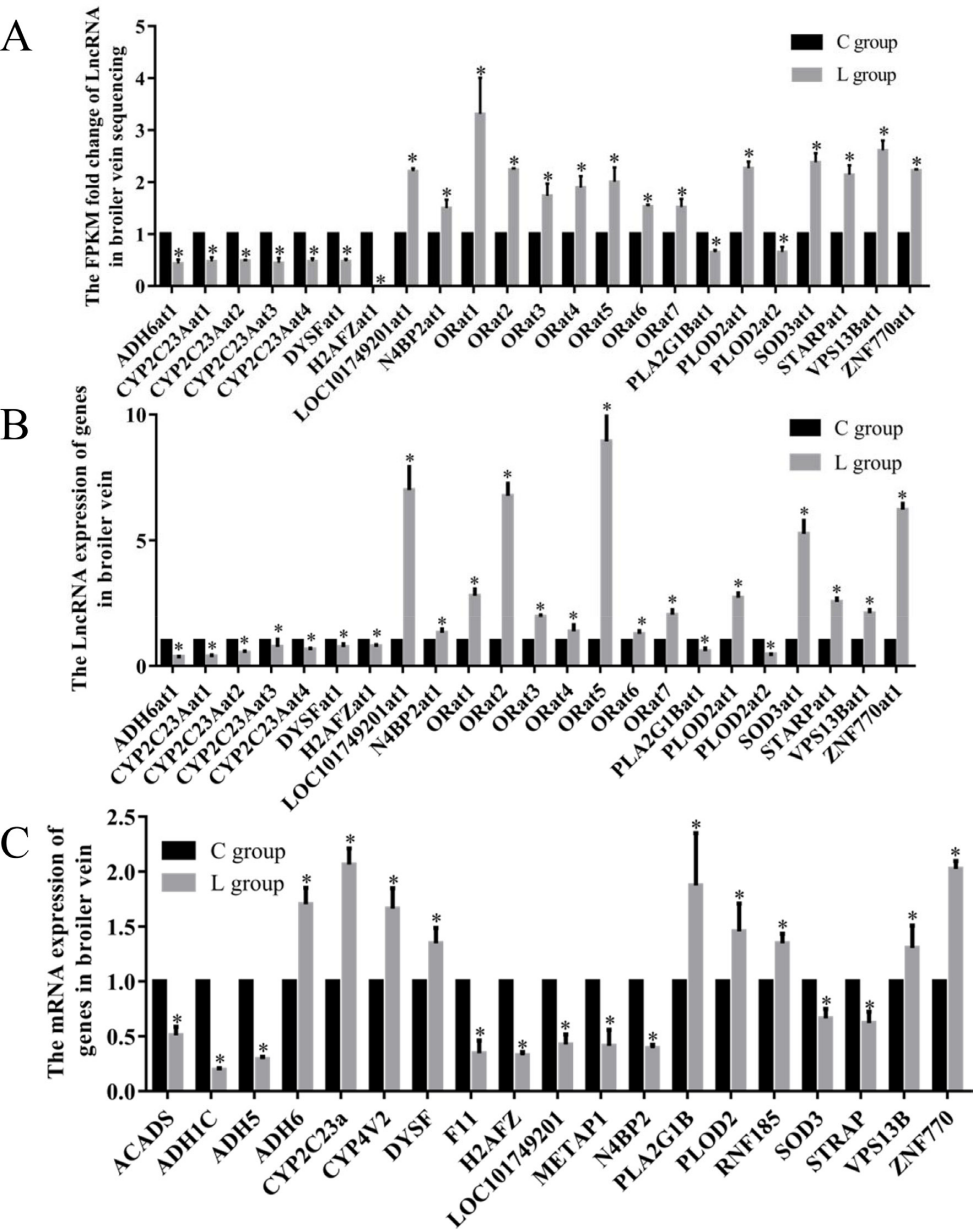
Effects of dietary Se level on the LncRNA and mRNA levels (**A**) The FPKM folds change of LncRNA in broiler chick vein sequencing; (**B**) Effects of dietary Se level on the LncRNA levels of vein in broiler chick. (**C**) Effects of dietary Se level on the relative mRNA levels of vein in broiler chick. Each value represented the mean ± S.D. of three individuals. *P < 0.05 versus control group.

In the significantly enriched GO term group, we filtered 19 target mRNAs related to oxidation reduction process term, the 19 selected mRNAs which neighbor 23 LncRNAs in 3 comparison groups. The RNA-seq results showed 23 LncRNAs were significantly changed by ED induced by Se deficiency. The LncRNA ID, target mRNA and abbreviation of LncRNA in this study are shown in Table 2. In this study, the FPKM fold change of LOC101749201at1, N4BP2at1, ORat1, ORat2, ORat3, ORat4, ORat5, ORat6, ORat7, PLOD2at1, SOD3at1, STARPat1, VPS13Bat1 and ZNF770at1 were actually 150–331% greater in the L group than the C group. ADH6at1, CYP2C23Aat1, CYP2C23Aat2, CYP2C23Aat3, CYP2C23Aat4, DYSFat1, H2AFZat1, PLA2G1Bat1 and PLOD2at2 were actually 34.3–99.5% lower in the L group than the C group (Figure 3A).

**Table 2:** The LncRNA ID, target mRNA and abbreviation of LncRNA

LncRNA_ID	Target mRNA_name	LncRNA_abbreviation
ALDBGALT0000000164	STRAP;RNF185	ORat1
ALDBGALT0000000166	STARP	STARPat1
ALDBGALT0000001763	ACADS;RNF185	ORat7
ALDBGALT0000002528	PLA2G1B	PLA2G1Bat1
ALDBGALT0000002790	VPS13B	VPS13Bat1
ALDBGALT0000003109	LOC101749201	LOC101749201at1
ALDBGALT0000004166	H2AFZ	H2AFZat1
ALDBGALT0000004189	N4BP2;CYP4V2	ORat2
ALDBGALT0000004204	ADH6	ADH6at1
ALDBGALT0000004205	SOD3	SOD3at1
ALDBGALT0000004423	ADH5;ADH1C;METAP1;ADH6	ORat3
ALDBGALT0000004424	H2AFZ;ADH1C	ORat4
ALDBGALT0000004426	H2AFZ;ADH1C;ADH6	ORat5
ALDBGALT0000004430	F11;H2AFZ;ADH1C	ORat6
ALDBGALT0000004441	N4BP2	N4BP2at1
ALDBGALT0000004504	DYSF	DYSFat1
ALDBGALT0000004595	ZNF770	ZNF770at1
ALDBGALT0000004930	CYP2C23A	CYP2C23Aat1
ALDBGALT0000004933	CYP2C23A	CYP2C23Aat2
ALDBGALT0000005048	CYP2C23A	CYP2C23Aat4
ALDBGALT0000005050	CYP2C23A	CYP2C23Aat3
ALDBGALT0000005584	PLOD2	PLOD2at1
ALDBGALT0000008087	PLOD2	PLOD2at2

### Validation of differential oxidative reduction-related LncRNAs in broiler vein by RT-PCR

To gain confidence in our transcript nominations, we validated 23 LncRNAs involved in oxidation reduction processes in poultry veins by RT-PCR (Figure 3B). These experiments revealed that 23 assayed genes in broiler veins were influenced (*P* < 0.05) by dietary Se. The Se deficiency broilers had decreased LncRNA levels of ADH6at1, CYP2C23Aat1, CYP2C23Aat2, CYP2C23Aat3, CYP2C23Aat4, DYSFat1, H2AFZat1, PLA2G1Bat1 and PLOD2at2 by 21–73% (*P* < 0.05). In contrast, vein LncRNA levels of LOC101749201at1, N4BP2at1, ORat1, ORat2, ORat3, ORat4, ORat5, ORat6, ORat7, PLOD2at1, SOD3at1, STARPat1, VPS13Bat1and ZNF770at1 were actually 128–893% greater (*P* < 0.05) in the L group than the C group.

### mRNA levels of genes related to oxidative stress in broiler vein by RT-PCR

Sequencing in cis way, we selected 19 mRNA which were co-localized with oxidative reduction-related LncRNA (Figure 3C). All 19 genes were affected by the dietary Se deficiency. Dietary Se deficiency resulted in a decrease (*P* < 0.05) in ACADS, ADH1C, ADH5, F11, H2AFZ, LOC101749201, METAP1, N4BP2, SOD3 and STRAP, but an increase (*P* < 0.05) in ADH6, CYP2C23A, CYP4V2, DYSF, PLA2G1B, PLOD2, RNA185, VPS13B and ZNF770, respectively.

### The LncRNA expressions in VECs

**Figure 4:**
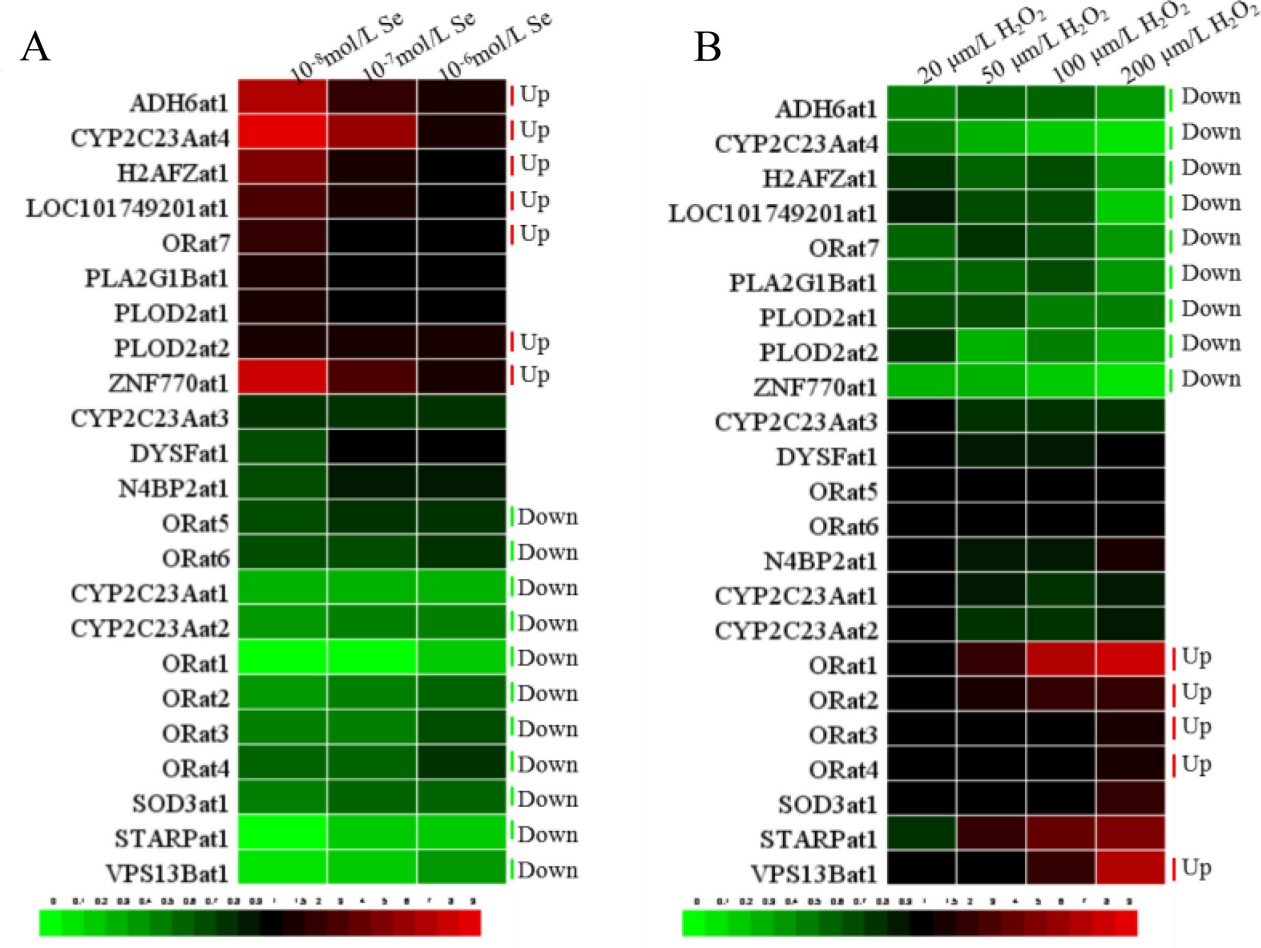
The heat-map of 23 LncRNAs in VECs (**A**) The heat-map of 23 LncRNAs in VECs when treated with Se (10^−8^, 10^−7^, 10^−6^ ml/L), (**B**) The heat-map of 23 LncRNAs in VECs when treated with H_2_O_2_ (20, 50, 100, 200 μm/L).

H_2_O_2_ contributes to oxidative stress, while Se can preserve the anti-oxidative function. To assess whether the expressions of the selected LncRNAs are regulated by oxidative stress, we exposed VECs to H_2_O_2_ and Se for 24 h. Heat maps of the levels of transcription of the 23 LncRNAs in H_2_O_2_ or Se-treated VECs are shown in Figure 4. To make the results clearer, we use a bar chart to display detailed information about each handle.

Inspection of the resulting diagram identified clusters of the 23 LncRNAs expression in the 10^−6^–10^−8^ m/L Se treated groups that appeared to show a similar effect (Supplementary Figure 1). These results revealed that the transcription of STARPat1, VPS13Bat1, CYP2C23Aat1, CYP2C23Aat2, SOD3at1, ORat1, ORat2, ORat3, ORat4, ORat5 and ORat6 were decreased (*P* < 0.05) compared with the C group. The transcription of PLOD2at1, ORat7, LOC101749201at1, H2AFZat1, ADH6at1, ZNF770at1 and CYP2C23Aat4 were increased (*P* < 0.05) compared with the C group.

Expression of LncRNA related to oxidative stress in VECs responded to H_2_O_2_ (20–100 μm/L) in different patterns (Supplementary Figure 2). The first was that H_2_O_2_ resulted in lower (*P* < 0.05) LncRNA levels compared with the C group. These decreases included PLOD2at1, LOC101749201at1, H2AFZat1, ADH6at1, ORat7, ZNF770at1 and CYP2C23Aat4. The second pattern had higher (*P* < 0.05) LncRNA levels compared to the C group. These up-regulations included CYP2C23Aat3, ORat1, ORat2, ORat3, ORat4, ORat5 and ORat6.

## DISCUSSION

Studies have shown the important role of LncRNA in regulating growth and development; and the function of LncRNA in regulating vascular health, VEC and VSMC has also been noticed [26–28]. This study uses Illumina Hi-Seq 4000 platform to sequence LncRNAs. In this study, we identified 15412 LncRNA transcripts from broiler vein including 8052 novel LncRNAs. There were 13085 LncRNA transcripts for chicken as shown in NONCODE database [29]. A total of 2626 LncRNAs were identified from two chicken lines either resistant or susceptible to Marek's Disease in CD4+ T cells on Illumina Hi-Seq 2000 [30].

ED is a classic Se deficiency disease [24]. ED may induce oxidative damage in broiler chick vascular tissue [31]. Oxidative stress is characterized by an increased level of reactive species, such as MDA and H_2_O_2_; cellular components such as Gpx and GSH may act as free radical scavengers [32, 33]. GPx is the key enzyme in the removal of H_2_O_2_ in biological systems while in turn, it needs GSH as a co-factor [34]. GSH is the primary antioxidant responsible for maintaining the reducing intracellular microenvironment that is essential for normal cellular function and viability. In this research, we found the activity of Gpx and GSH was down-regulated by a Se deficient diet, which indicated the reduced antioxidant capacity in vein may have functional consequences in terms of vascular damage, eventually leading to the occurrence of ED.

Pathological changes in broiler chicks are powerful indicators of vein damage in ED induced by Se deficiency. The aberrant VECs and VSMCs may disturb the organism's normal metabolism such as the oxidative reduction process, thereby inducing ED [35]. The morphological findings in this study indicated a series of lesions in broiler vein tissues that demonstrated the severe damage of Se deficiency which would ultimately influence the normal oxidative reduction process and the incidence of ED.

Compared with the control broiler chicks, we found 635 LncRNAs significantly changed in ED induced by Se deficiency. We also enriched the LncRNAs to 22 main terms. The result showed the potential roles of these LncRNAs in regulating ED. In the 22 terms, the LncRNAs involved in carbohydrate metabolic processes were the most predominant. The LncRNAs enriched in the oxidation-reduction process term was the second. The carbohydrate metabolic process may be involved in many reactions. The change of diet and rearing environment may influence the carbohydrate metabolic process [36, 37]. Many studies have demonstrated the roles of Se in regulating the oxidative status in different tissues and cells [17, 18, 38, 39]; oxidative stress has also been found in arteries and veins. Because of the important role of the oxidative reduction process in ED, this experiment focused on the LncRNAs related to the oxidative reduction process. The change in these 23 LncRNAs demonstrates their essential role in the ED process.

In the 23 LncRNAs related to oxidative stress, 14 LncRNAs were up-regulated and 9 LncRNAs were down-regulated. We hypothesized that the up-regulated LncRNA performed an antioxidant role while the down-regulated LncRNAs were involved in the oxidative-reduction process. Studies showed that LncRNA may regulate the oxidation-reduction level [8, 9]. We verified the 23 significantly changed LncRNAs by RT-PCR *in vivo*. These results could confirm that our resultant LncRNAs were of high quality.

In this study, LncRNAs associated oxidative reduction-related 19 mRNA were detected. Our results demonstrated that the deficiency of Se may inhibit 10 and promote 9 genes’ expression by RT-PCR. ACADS is bona fide peroxisomal proteins in mammals. It belongs to the basic enzymatic repertoire of peroxisomes [40]. ADH5 and ADH6 can also metabolize lipid peroxidation products [41]. Se has the ability to protect genes against the imbalance of lipid peroxidation products. Epidemiologic investigations showed correlations between abnormal lipid metabolism and decreased plasma Se concentrations [42]. Seale et al. showed that knockout of selenocysteinelyase in mice affected lipid homeostasis [43]. In the present study, the expression of ACADS, ADH5 showed decreased tendencies, indicating the imbalance of lipid metabolism, which in turn influences the oxidation-reduction reaction. ADH1C, F11, STRAP, VPS13B, SOD3 and METAP1 may be particularly responsible for oxidation induced by various stimulations [44–47]. SOD is especially responsible for preventing the formation of free radicals [23]. SOD3 belongs to the primary antioxidant defense system of the organism whose role is mainly preventive against oxidative stress. Two other important factors influencing the redox function in tissue were CYP2C23A and CYP4V2. They encode members of the cytochrome P450 hemethiolate protein superfamily, which are involved in oxidizing various substrates [48]. It has been reported the distribution of CYP450 in hepatic tissues were regulated by Se [49]. In this study, the mRNA expression of CYP2C23A and CYP4V2 was up-regulated by Se deficiency; the mechanism might be the extra generation of cytochrome P450 protects tissues against peroxide.

In the *in vitro* model, the 23 LncRNAs related to oxidative stress showed similar adverse trends with the sequencing result. H_2_O_2_ can induce oxidative stress in cells [50]. However, Se play adverse role in the oxidative reduction process. Se is involved in several metabolic processes, the most important as an antioxidant protecting the body against oxidative damage. ED disease ultimately leads to the deficiency of Se-containing internal antioxidants, while supplementation of Se leads to enhanced comparative metabolism in cells [51, 52]. A previous study indicated that the anti-oxidation properties of cells was improved with increasing Se doses [38]. In this study, we used Se and H_2_O_2_ to establish an oxidative stress model of vein VECs to detect the change in oxidative reduction-related LncRNAs *in vitro*. With rising concentrations of H_2_O_2_, the oxidative-reduction balance was disrupted and oxidative stress was enhanced in cells. The high concentration of H_2_O_2_ had a wider influence on the LncRNAs related to oxidative stress. These results demonstrated that LncRNAs do play roles in oxidative stress. In this present study, the selected LncRNAs with Se treatment groups showed a contrary trend with the H_2_O_2_ treatment groups. As the Se and H_2_O_2_ antagonized each other, the results of 23 LncRNA confirmed this. This makes the LncRNAs that participate in the oxidative reduction process more certain.

In conclusion, this study provides a complete LncRNA transcriptome profile in broiler chick vein for the first time, and analyzes the change of LncRNAs in vein damage caused by ED. 15412 LncRNAs were detected in this study and 635 LncRNAs were significantly changed. The 23 significantly changed LncRNAs were involved in the oxidative reduction process. This study showed that LncRNAs participated in the oxidative reduction process in broiler vein, predicting the mechanism in ED. In addition, our ongoing effort will focus on the function of some LncRNAs through experimental approaches, expecting to provide more fundamental information in understanding their regulatory mechanisms of oxidative stress induced by Se deficiency at the molecular level.

## MATERIALS AND METHODS

### Birds and diets

All of the procedures used in this study were approved by the Institutional Animal Care and Use Committee of the Northeast Agricultural University. 60 male broiler chicks (1 day old; Weiwei Co. Ltd., Harbin, China) were randomly divided into two groups (30 broilers per group). The broilers were fed either a commercial granulated diet (C group, with a final Se content of 0.2 mg/kg) or a Se-deficient granulated diet (L group, from the Se deficient region of Heilongjiang Province in China, containing 0.008 mg/kg). Food and water were provided ad libitum. Following euthanasia with sodium pentobarbital, veins were quickly removed, blotted, rinsed with ice-cold sterile deionized water, frozen immediately in liquid nitrogen, and stored at −80°C until required.

### Determination of antioxidant ability

The protein content was measured using the protein quantitative detection kit (A045-2, Nanjing Jiancheng Bioengineering Institute, P.R. China) according to the manufacturer's protocol. The GPx activity, content of GSH, MDA and H_2_O_2_ were measured using kit (A005, A004, A003, A064, Nanjing Jiancheng Bioengineering Institute, Nanjing, China) according to the manufacturer's protocol.

### Histopathological examination

Vein and skin tissues were fixed in 10% formaldehyde and embedded in paraffin for microscopic examination. Sections (5 μm thick) were cut and stained with hematoxylin and eosin (H&E) and examined under microscope by a pathologist in a blinded manner.

### RNA isolation

RNA degradation and contamination was monitored on 1% agarose gels. RNA purity was checked using the NanoPhotometer^®^ spectrophotometer (IMPLEN, CA, USA). RNA concentration was measured using Qubit^®^ RNA Assay Kit in Qubit^®^ 2.0 Flurometer (Life Technologies, CA, USA). RNA integrity was assessed using the RNA Nano 6000 Assay Kit of the Bioanalyzer 2100 system (Agilent Technologies, CA, USA).

### Library preparation for LncRNA sequencing

A total amount of 3 μg RNA from 3 vein tissues was used as input material for the RNA sample preparations. First, ribosomal RNA was removed by EpicentreRibo-zero™ rRNA Removal Kit (Epicentre, USA) and rRNA free residue was cleaned up by ethanol precipitation. Subsequently, sequencing libraries were generated using the rRNA-depleted RNA by NEBNext^®^ Ultra^™^ Directional RNA Library Prep Kit for Illumina^®^ (NEB, USA) following the manufacturer's recommendations. Finally, products were purified (AMPure XP system) and library quality was assessed on the Agilent Bioanalyzer 2100 system.

### Clustering, sequencing and target gene prediction

The clustering of the index-coded samples was performed on a cBot Cluster Generation System using TruSeq PE Cluster Kit v3-cBot-HS (Illumia) according to the manufacturer's instructions. After cluster generation, the libraries were sequenced on an Illumina Hi-seq 4000 platform and 100 bp paired-end reads were generated. Transcripts without coding potential were our candidate set of LncRNAs. Then, we searched coding genes 10k/100k upstream and downstream of filtered differentially expressed LncRNA as the cis target genes.

### GO enrichment analysis

To understand the function roles of the target genes of LncRNA, we used the GOseq R package to implement an enrichment analysis in which gene length bias was corrected. GO terms with corrected *P value* of less than 0.05 were considered significantly enriched by differentially expressed target genes of LncRNA.

### Primary cultures of broiler vein endothelial Cells (VECs) and treatments

Broiler vein primary endothelial cells (VECs) were isolated using collagenase II (Sigma-Aldrich, St. Louis, MO) and cultured in Medium 199 (Invitrogen) with 20% (v/v) fetal bovine serum (FBS, Invitrogen) and 100 U/ml penicillin, 100 μg/ml streptomycin 37°C in a 5% CO_2_ and 95% air atmosphere. For the monitoring of various parameters in the present investigation, cells were treated for 24 h in the presence of various concentrations of H_2_O_2_ (20, 50, 100, 200 μm/L), and incubated with 10^−8^, 10^−7^ and 10^−6^ m/L of Se as Sodium Selenite (Sigma, USA) for 24 h.

### Real-time quantitative PCR analysis on mRNA and LncRNA levels

Total RNA was extracted from vein tissues and cell samples and the complementary DNA was synthesized using a RevertAid first strand cDNA synthesis kit (Thermo Scientific, MA, USA). Detected via qRT-PCR; gene expression levels were performed on a Light Cycler^®^ 480 System (Roche, Basel, Switzerland) using Fast Universal SYBR Green Master (Roche, Basel, Switzerland). Primer Analysis Software (Oligo 7.24, Molecular BiologyInsights, Inc., USA) was used to design specific oligonucleotide primers. These mRNA and LncRNA primers were commercially synthesized by Beijing Genomics Institute Co., Ltd., China. GADPH was the housekeeping gene used as an internal reference. The mRNA and LncRNA relative abundance for each gene was calculated according to the method of 2^−ΔΔCt^, accounting for gene specific efficiency and was normalized to the mean expression of GADPH.

### Statistical analysis

GraphPad Prism 7.0 (GraphPad Software Inc., USA) and Microsoft Office Excel 2010 were used to test the effects of the dietary Se levels on measures. Multiple mean comparisons were performed using One-way ANOVA. Data are presented as the means ± S.D. and values were considered statistically significant if *P* < 0.05. Ranking of genes by the degree of differential expression was analyzed with a heat map using the Heml 1.0 (http://hemi.biocuckoo.org/down.php).

## SUPPLEMENTARY MATERIALS FIGURES


