# Supplementary material for: Impact of exudative diathesis induced by selenium deficiency on LncRNAs and their roles in the oxidative reduction process in broiler chick veins
Source: Oncotarget. 2017 Feb 1;8(13):20695–705. doi: 10.18632/oncotarget.14971 (PMC5400537; doi:10.18632/oncotarget.14971)
Supplement: Supplementary file 1 [file oncotarget-08-20695-s001.pdf]

Impact of exudative diathesis induced by selenium deficiency on LncRNAs and their roles in the oxidative reduction process in broiler chick vein

Supplementary Materials

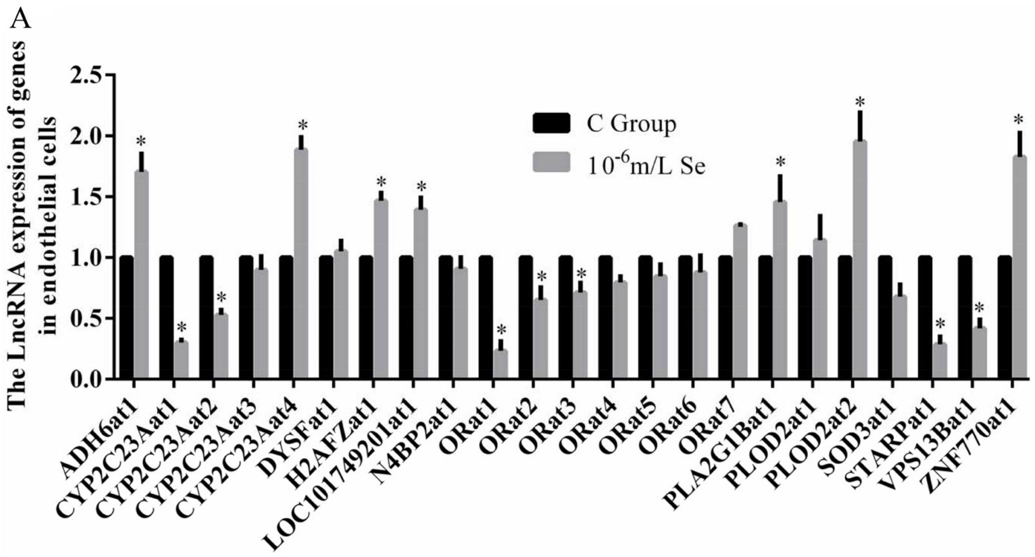

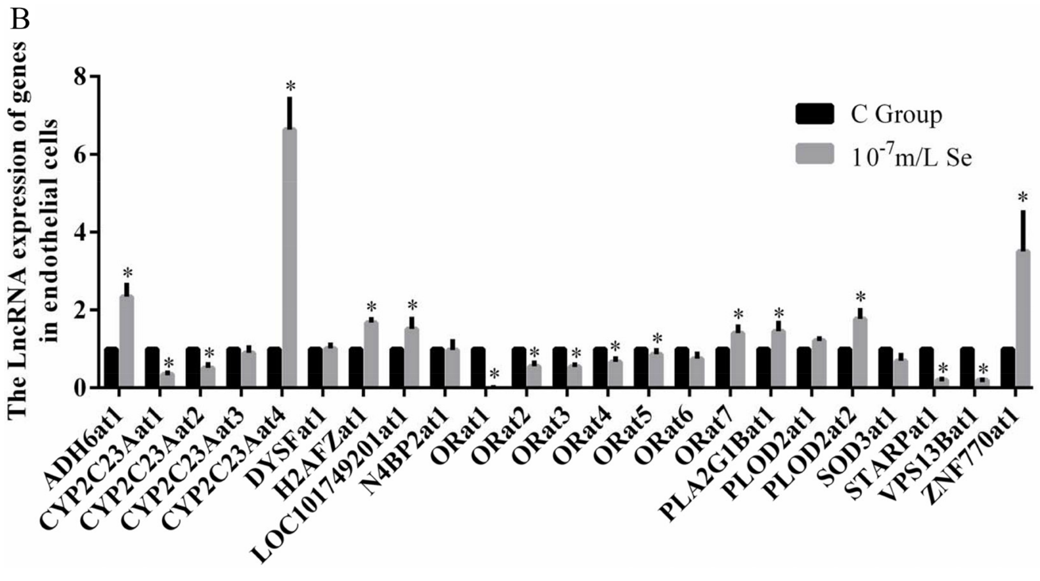

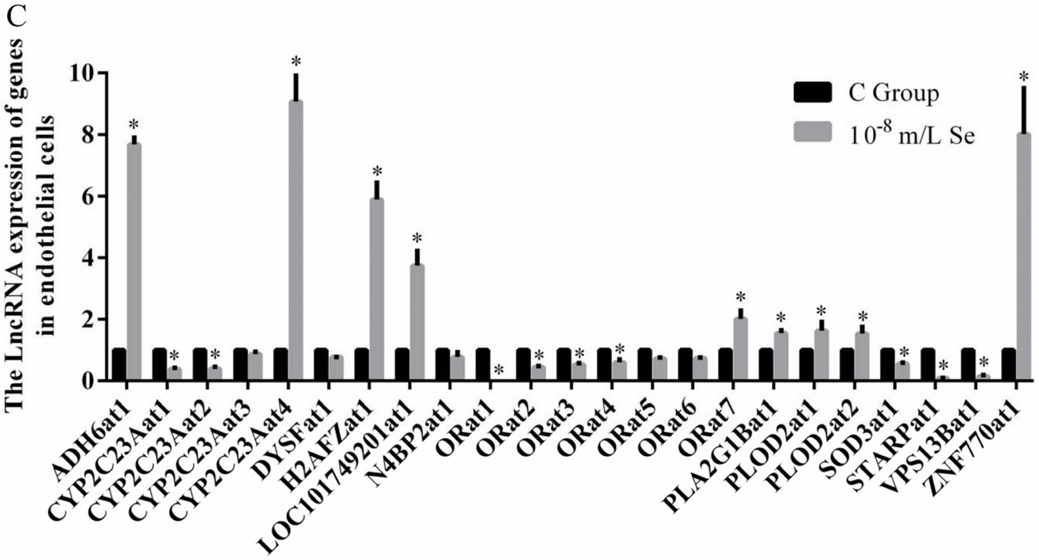

Supplementary Figure 1: Effects of Se on the relative mRNA levels in VECs. (**A**) 10^{-6} m/L Se treated; (**B**) 10^{-7} m/L Se treated; (**C**) 10^{-8} m/L Se treated.

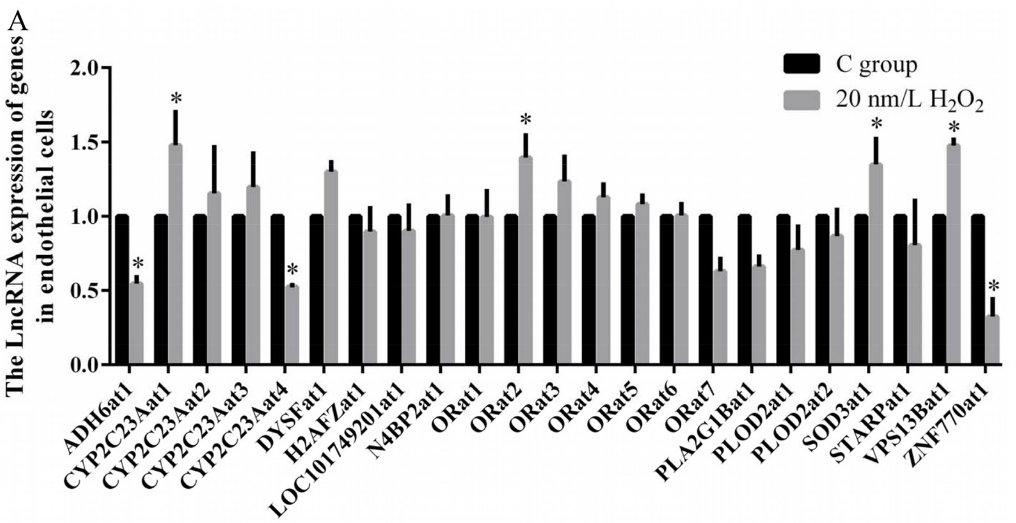

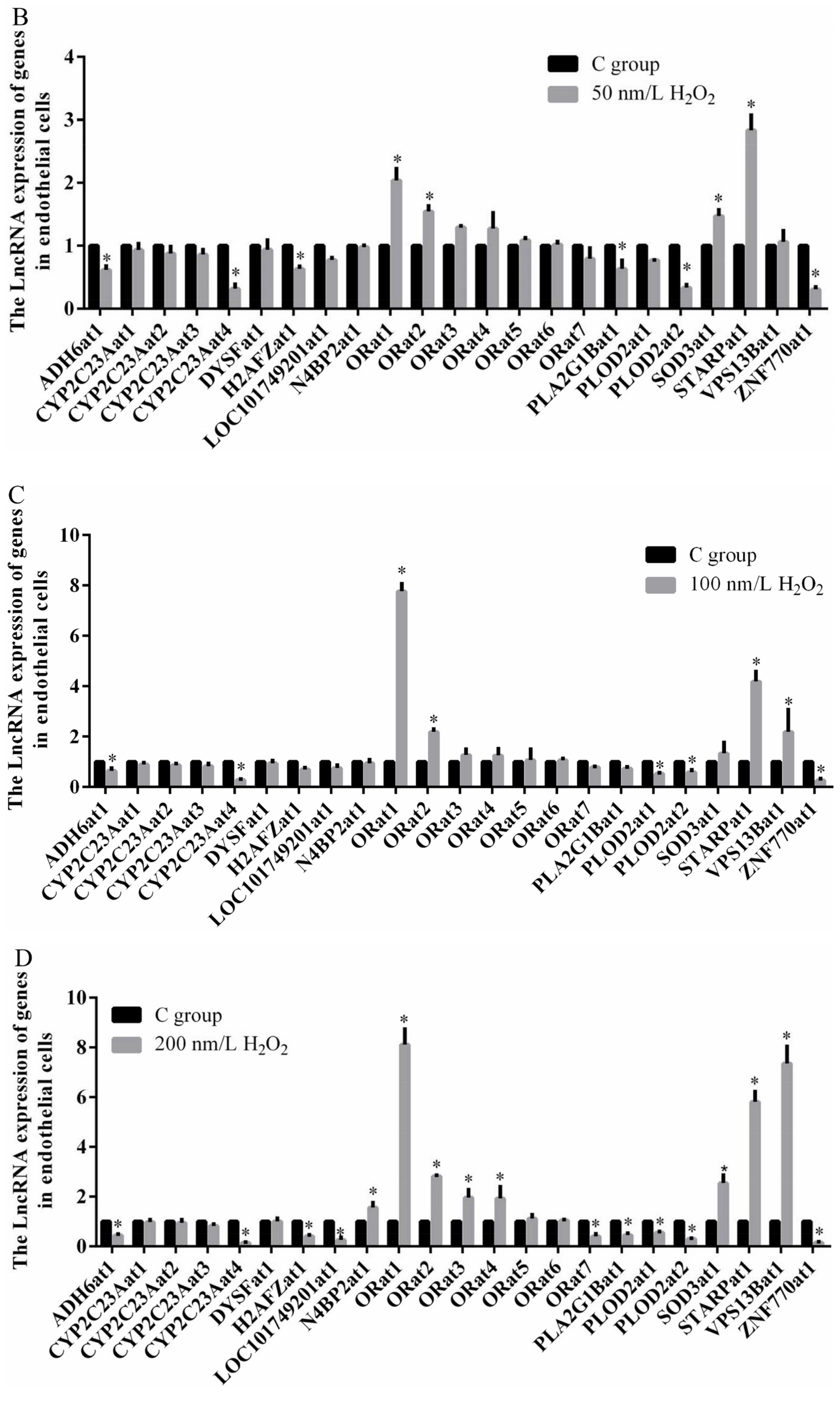

Supplementary Figure 2: Effects of H_2O_2 on the relative mRNA levels in VECs. (**A**) 20 nm/L H_2O_2 treated; (**B**) 50 nm/L H_2O_2 treated; (**C**) 100 nm/L H_2O_2 treated; (**D**) 200 nm/L H_2O_2 treated.
